# High-fat, high-carbohydrate diet-induced prediabetes preconception in Sprague–Dawley rats as a risk factor for the development of preeclampsia: assessing changes in placental metabolic insults

**DOI:** 10.3389/fnut.2023.1241785

**Published:** 2023-10-23

**Authors:** Asiphaphola Ludidi, Anelisiwe Siboto, Ayanda Nkosi, Nombuso Duduzile Xulu, Andile Khathi, Ntethelelo Hopewell Sibiya, Phikelelani Siphosethu Ngubane

**Affiliations:** ^1^School of Laboratory Medicine and Medical Sciences, College of Health Sciences, University of KwaZulu-Natal, Durban, South Africa; ^2^Division of Pharmacy, Rhodes University, Makhanda, South Africa

**Keywords:** hyperglycemia, diet, inflammation, placenta, prediabetes, preeclampsia

## Abstract

**Introduction:**

Hyperglycemia preconception deranges the establishment of a functional placenta; however, the risk of developing preeclampsia (PE) in prediabetic patients remains obscure. The aim was to assess abnormal placental changes as a risk factor for the development of PE in high-fat, high-carbohydrate (HFHC) diet-induced prediabetic (PD) rats.

**Methods:**

HFHC diet-induced female prediabetic Sprague–Dawley rats were mated, and blood glucose concentrations, mean arterial pressure (MAP), and body weights were monitored on gestational days (GNDs) 0, 9, and 18. On GND 18, animals were euthanized. Blood and placentas were collected for biochemical analysis.

**Results:**

Prediabetic rats showed significantly increased blood glucose concentration, proinflammatory cytokines, MAP, placental weight, and fetoplacental ratio compared with non-prediabetic (NPD) rats. Prediabetic rats showed significantly decreased placental vascular endothelial growth factor receptor 1 (VEGFR1) and placental growth factor (PLGF) and plasma nitric oxide (NO) compared with NPD.

**Discussion:**

Prediabetes may have promoted endothelial dysfunction in the placenta and hypoxia, thus reducing PLGF and VEGFR1, which may have promoted proinflammation, endothelial dysfunction associated with NO decline, and hypertension, which is also observed in preeclamptic patients. Prediabetes may have promoted lipogenesis in placentas and fetuses that may have induced macrosomia and IUGR, also observed in preeclamptic patients. The findings from this study highlight the need for screening and monitoring of prediabetes during pregnancy to reduce the risk of developing preeclampsia.

## 1. Introduction

Poor glycemic control is associated with insulin resistance (IR) and a proinflammatory state of diabetes mellitus (DM) preconception, which increases the risk of developing pregnancy-related complications such as preeclampsia (PE) that may develop eclampsia (1, 2). However, there is limited literature that has reported the risk of the development of PE in prediabetic patients. PE significantly increases the risk of preterm delivery, macrosomia, miscarriage, and death of the pregnant mother, fetus, and/or both (3, 4). PE is a pregnancy-related hypertensive disorder that affects 3–6% of pregnant women (5). Clinically, PE is characterized by an elevated blood pressure of above 140/90 mmHg and proteinuria of over 300 mg/day, which is also observed in diabetic patients (6, 7). PE is the leading cause of maternal mortality in low- to middle-income countries and has been estimated to affect 2–10% of pregnant women annually (8, 9). The etiology of PE is not fully understood; however, the literature suggests that the dysregulation of placentogenesis due to a compromised uterine wall preconception, as observed in diabetic patients, may play a significant role in the pathology of PE (10, 11). The embryo may embed itself in the compromised uterine wall, resulting in inadequate trophoblast invasion and migration, shallow implantation of the placenta, and reduced vascularization and spiral artery remodeling, hence establishing a malformed placenta (12). The malformed placenta has been shown to have reduced placental perfusion due to defective spiral artery remodeling and increased placental ischemia (13). The hypoxic placenta has been shown to be associated with reduced production and expression of proangiogenic factors, such as placental growth factor (PLGF) and vascular endothelial growth factor (VEGF) and their receptors including the VEGF receptor 1/Fms-like tyrosine kinase-1 (VEGFR1/Flt-1), which alter angiogenesis and vascular homeostasis (14, 15). Furthermore, there is an increased production of antiangiogenic factors including soluble fms-like tyrosine kinase-1 (sFlt-1), which is a non-signaling decoy VEGFR1 receptor, soluble endoglin (sEng), and proinflammatory cytokines, such as interferon gamma (IFN- γ), tumor necrosis factor alpha (TNFα), and interleukin-6 (IL-6) (16–18). The antiangiogenic factors and proinflammatory cytokines then enter the maternal circulation and initiate widespread maternal endothelial dysfunction, which is associated with a reduction in the production and bioavailability of the vasodilator, nitric oxide (NO) which is associated with hypertension that is also observed in preeclamptic patients (19). Previous rat model studies have shown that the administration of the NO synthase inhibitor, N(omega)-nitro-L-arginine methyl ester (L-NAME), resulted in pathological changes that are similar to those observed in preeclamptic patients (20, 21). In addition, hyperglycemia in animal models has been shown to elevate proinflammatory cytokine secretion and apoptosis in trophoblasts which may be associated with the shallow implantation of the placenta, thus contributing to the risk of developing PE in diabetic patients (22). However, the mechanisms that increase the risk of developing PE in a prediabetic state have not been investigated. Therefore, this study aimed to assess the changes in placental metabolic insults of prediabetic animals to investigate the risk of developing PE-related complications.

## 2. Materials and methods

### 2.1. Drugs and chemical reagents

All the drugs and chemicals were sourced from standard pharmaceutical suppliers. Commercial suppliers provided chemicals of analytical grade, such as glucose (C_6_H_12_O_6_), sodium nitrite, vanadium chloride (VCl_3_), sulphanilamide (SULF), *N*-1-napthylethylenediamine dihydrochloride (NEDD) (Merck chemicals (Pty) Ltd. Wadeville, Johannesburg, South Africa), N(omega)-nitro-L-arginine methyl ester, and phosphate-buffered saline (PBS) (Sigma–Aldrich Co., St. Louis, MO).

### 2.2. Animals

Eighteen 3-week-old female Sprague–Dawley rats (150–180g) were used in the present study. The rats were bred and housed in the Biomedical Research Unit (BRU) of University of KwaZulu-Natal. The animals were maintained under the following conditions: under standard laboratory conditions of constant temperature (22 ± 2°C), CO_2_ content of >5,000 p.m., relative humidity of 55 ± 5%, illumination (12 h light/dark cycles), noise levels of < 65 decibels, and *ad libitum* access to food and water. All the procedures involving animals and their care were conducted according to the institutional guidelines of the University of KwaZulu-Natal (AREC/031/019D). Rats were housed in standard conventional polycarbonate 1291H tecniplast cages and were acclimatized for 5 days prior to commencement of the study.

### 2.3. Induction of prediabetes

The animals were randomly assigned to the following experimental groups: a standard diet with normal drinking water (*n* = 12) and a high-fat, high-carbohydrate diet with drinking water supplemented with 15% fructose (HFHC, *n* = 6) [AVI Products (Pty) Ltd., Waterfall, South Africa]. The animals were allowed to consume the HFHC diet *ad libitum* for 9 months to induce prediabetes using a previously described protocol (23, 24). After 9 months, the American Diabetes Association (ADA) criteria were used as a guideline to diagnose prediabetes. It defines prediabetes as a fasting plasma glucose (FPG) concentration between 5.6 and 6.9 mmol/L and impaired glucose tolerance (IGT) between 7.8 and 11.0 mmol/L during an oral glucose tolerance test (OGTT). Prediabetic rats continued with the experiment.

### 2.4. Mating

Before mating, all 18 female Sprague–Dawley rats were assessed for the proestrus stage using the vaginal smear, which was analyzed under the microscope. Animals that were in the proestrus stage were allowed to mate with healthy male Sprague–Dawley rats. In the next morning, the presence of a vaginal plaque or vaginal smear with the presence of spermatozoa after observing under a microscope was used to confirm pregnancy and assigned gestational day (GND) 0. The male rat was removed from the cage after successful mating (25). Pregnant rats continued with the experiment until gestational day 18.

### 2.5. Experimental protocol

The effects of prediabetes on the risk of the development of preeclampsia in Sprague–Dawley rats over an experimental period of 39 weeks were investigated. The animals were randomly assigned to the following experimental groups: standard diet with normal drinking water (NPD), a high-fat, high-carbohydrate diet with drinking water supplemented with 15% fructose (PD), and a standard diet with L-NAME in drinking water (PE). The preeclamptic group consumed 0.3 g/L of N^ω^-nitro-l-arginine methyl ester (L-NAME) in drinking water a day from GND 8 to 18. Mean arterial blood pressure (MAP) and blood glucose concentration were monitored over the gestational experimental period. MAP was measured using the non-invasive tail-cuff method with photoelectric sensors (IITC Model 31 Computerized Blood Pressure Monitor, Life Sciences, Woodland Hills, California, USA) as previously described (26), while the tail-prick method was applied to measure blood glucose concentrations utilizing the Elite^®^ glucometer [Elite (Pty) Ltd., Health Care Division, South Africa] at 09:00 on GND 0, 9, and 18.

### 2.6. Tissue sample harvesting

On GND 18, all 18 female animals were euthanized. The blood was then collected in individual pre-cooled heparinized tubes and centrifuged (Eppendorf centrifuge 5403, Germany) at 4°C and 503 g for 15 min. Then, plasma was separated and stored at −80°C in a BioUltra freezer (Snijers Scientific, Holland) for hormonal and biochemical analyses. The placental tissues were removed, weighed, snap frozen in liquid nitrogen, and then stored at −80°C in a BioUltra freezer until use (Snijers Scientific, Tilburg, Netherlands).

### 2.7. Biochemical analysis

#### 2.7.1. Oral glucose tolerance test

An OGTT was performed on all 18 female rats on GND 0, 9, and 18 as previously described (27).

#### 2.7.2. Determination of IFN- γ, PLGF, and VEGFR1 concentration

Placental PLGF, VEGFR1, and IFN- γ concentrations were analyzed using a separate specific sandwich ELISA kit (Elabscience Biotechnology Co., LTD., Wuhan), according to the guidelines in the manufacturer's manual.

#### 2.7.3. Determination of placental TNFα and IL-6 gene expression

##### 2.7.3.1. Real-time polymerase chain reaction (RT-PCR)

Approximately 50 mg of the placental tissue was weighed (*n* = 6 per group), homogenized, and suspended in 400 μl of lysis buffer (Zymo Research, USA). Thereafter, samples were homogenized using a sonicator, and the total RNA was isolated according to the manufacturers' guidelines (ZR RNA MiniPrepTM, USA). Purification of RNA isolates was determined using a NanoDrop spectrophotometer (Thermo Scientific, Johannesburg, South Africa). A purity of 1.5–2.01 (A260/280 nm) was considered ideal for use in the synthesis of cDNA. cDNA was synthesized using the iScript™ cDNA Synthesis Kit (Biorad, South Africa), according to the manufacturers' guidelines. cDNA was run through the thermocycler as per the conditions present in the guidelines. The FastStart SYBR Green Kit (Roche Diagnostics, USA) was used according to the manufacturer's protocol. TNFα and IL-6 gene expression was determined using oligonucleotide primers (Table 1) which were reconstituted in RNA nuclease-free water, according to the manufacturer's guidelines (Inqaba Biotechnical Industries, South Africa). In total, 10 μl reaction volume was prepared which contained 5 μl of SYBR Green Master Mix, 1 μl of the forward primer, 1 μl of the reverse primer, 2 μl of the cDNA template, and 1 μl of nuclease-free H_2_O. The reaction mix was then incubated in a light cycler 96 RT-PCR system (Roche, Mannheim, Germany) at optimized conditions: initial denaturation cycle at 95°C for 10 min followed by 45 cycles at 95°C for denaturation for 15 s, annealing at 60°C for 60 sec, and elongation at 72°C for 20 s. Melting curves were generated at the end of each PCR, and the Lightcycler^®^ analysis software (version 4.2) was used to analyze and verify the purity and specificity of the amplified PCR products. The 2^−Δ*Δct*^ method was used to calculate the relative mRNA expression and normalized in relation to the housekeeping gene glyceraldehyde-3-phosphate (GAPDH) (28).

**Table 1:** Oligonucleotide primers used in experiments.

**Name of gene**	**Sequence (5^′^to 3^′^)**
IL-6	Forward: 5′ GTTGTGCAATGGCAATTCTGA 3′
	Reverse: 5′ TCTGACAGTGCATCATCGCTG 3′
TNFα	Forward: 5′ TCCAATGGGCTTTCGGAAC 3′
	Reverse: 5′ CACTCAGGCATCGACATTCG 3′
GAPDH	Forward: 5′ CTC TAC CCA CGG CAAGTT CAA 3′
	Reverse: 5′ GGA TGA CCT TGC CCA CAGC 3′

#### 2.7.4. Determination of plasma nitric oxide concentration

Approximately 25 μl of the plasma was used to assess NO concentration in the samples followed by a previously described protocol (29).

### 2.8. Statistical analysis

All the data were expressed as means ± standard deviation (SD). GraphPad Prism InStat Software (version 5.00, GraphPad Software, San Diego, California, USA) was used to perform statistical analyses. All the data were tested for normality using the Kolmogorov–Smirnov and Shapiro–Wilk tests. A two-way analysis of variance (ANOVA) test was used to further analyze the differences in the results between the control and experimental groups, followed by Tukey's *post-hoc* test. Values of *p* < 0.05 were regarded as statistically significant between the compared groups.

## 3. Results

### 3.1. Blood glucose concentration

**Figure 1:**
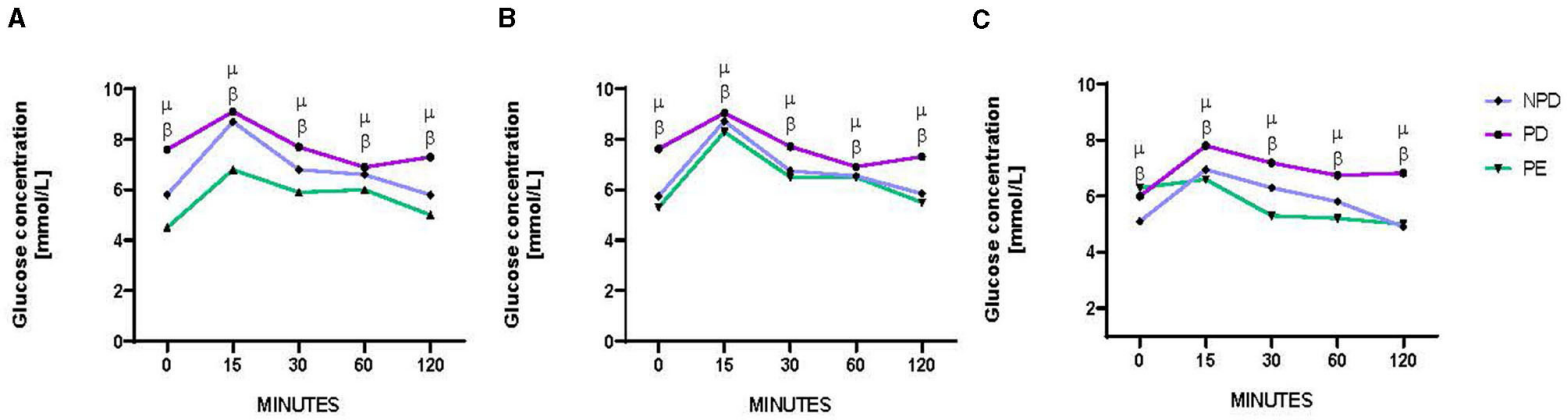
Blood glucose concentration. Comparison of oral glucose tolerance in NPD, PD and PE animals at GND 0 **(A)**, GND 9 **(B)**, GND 18 **(C)**. Values are presented as means and vertical bars indicate SD (*n* = 6 in each group). ^β^*p* < 0.05 vs. NPD and ^μ^*p* < 0.05 vs. PD.

Figure 1 shows the results of the oral glucose tolerance test (OGTT) of non-prediabetic (NPD), prediabetic (PD), and preeclamptic (PE) animals at GND 0 (Figure 1A), GND 9 (Figure 1B), and GND 18 (Figure 1C). A significant difference was observed between the experimental groups, as shown in Figure 1A [F_(2, 8)_ = 86.22, *p* = 0.0004], Figure 1B [F_(2, 8)_ = 12.83, *p* = 0.0032], and Figure 1C [F_(2, 8)_ = 18.81, *p* = 0.0009]. At the beginning of the OGTT, fasting glucose was measured at 0 min, and the PD group was found to have a significantly higher blood glucose concentration at 0 min on GND 0 (Figure 1A), 9 (Figure 1B), and 18 (Figure 1C) than the NPD group ^β^ (NPD vs. PD, *p* < 0.05, Figure 1). In addition, the PD group had a significantly higher blood glucose concentration at 0 min on GND 0 (Figure 1A) and 9 (Figure 1B) than the PE group ^μ^ (PD vs. PE, *p* < 0.05, Figure 1). After glucose administration, the PD group had a significantly higher blood glucose concentration throughout the 120 min on GND 0 (Figure 1A), 9 (Figure 1B), and 18 (Figure 1C) than the NPD group ^β^ (NPD vs. PD, *p* < 0.05, Figure 1). Furthermore, after glucose administration, the PD group had a significantly higher blood glucose concentration throughout the 120 min on GND 0 (Figure 1A), 9 (Figure 1B), and 18 (Figure 1C) than the PE group ^μ^ (PD vs. PE, *p* < 0.05, Figure 1).

### 3.2. Mean arterial pressure

**Figure 2:**
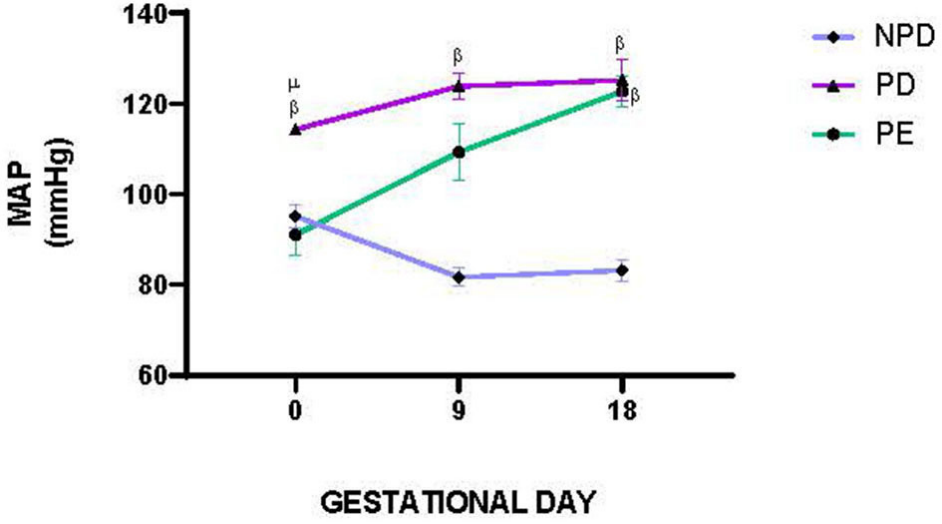
Mean arterial pressure. Comparison of MAP in NPD, PD and PE animals at GND 0, 9 and 18. Values are presented as means and vertical bars indicate SD (*n* = 6 in each group). ^β^*p* < 0.05 vs. NPD and ^μ^*p* < 0.05 vs. PD.

Figure 2 shows the mean arterial pressure (MAP) of NPD, PD, and PE animals at GND 0, 9, and 18. A significant difference was observed between the experimental groups [F_(2, 5)_ = 593.4, *p* = 0.0001]. The PD group had a significantly lower MAP than the NPD group ^β^(NPD vs. PD, *p* < 0.05, Figure 2). Furthermore, the PD group had a significantly higher MAP than the PE group ^μ^ (PD vs. PE, *p* < 0.05, Figure 2).

### 3.3. Placenta to body weight and pup weight ratio

Table 2 compares the relative placental weight and fetoplacental ratio at the end of the experimental period in NPD, PD, and PE animals. The experimental groups showed significantly different relative placental to body weight [F_(2, 10)_ = 4.054, *p* = 0.0145] and fetoplacental ratios. The PD group had a slightly increased placental to body weight and a significantly higher fetoplacental ratio than the NPD group ^β^ (NPD vs. PD, *p* < 0.05). Furthermore, the PD group had a significantly increased placental weight and higher fetoplacental ratio than the PE group ^μ^ (PD vs. PE, *p* < 0.05, Table 2).

**Table 2:** Comparison of relative placental weight and foetoplacental ratio at the end of the experimental period in NPD, PD and PE animals.

**Experimental groups**	**Relative placental weight ratio**	**Foetoplacental ratio**
NPD	0.25 ± 1.08	0.16 ± 0.09
PD	0.27 ± 0.55 μ	0.32 ± 0.25 β
PE	0.2 ± 0.24 β	0.24 ± 0.21 β

Values are presented as means and vertical bars indicate SD (*n* = 6 in each group).

### 3.4. Placental PLGF and VEGFR1 concentration

**Figure 3:**
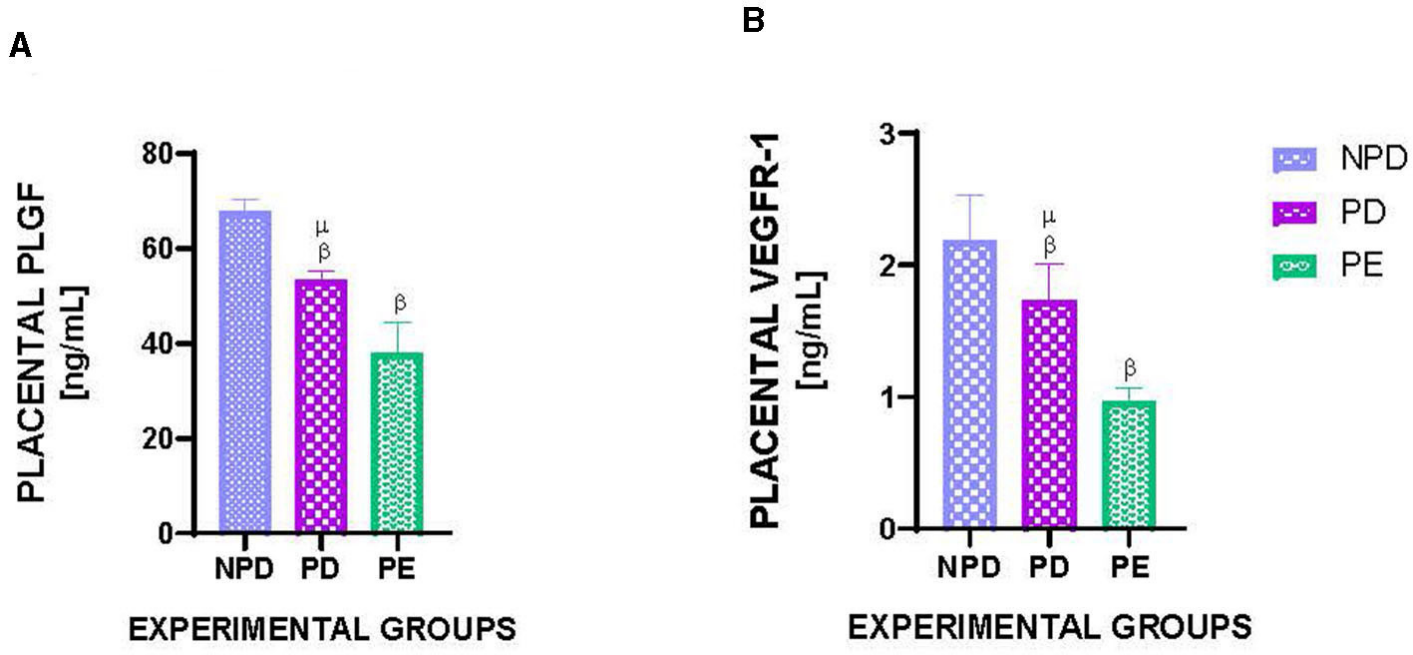
Placental PLGF and VEGFR1 concentration. Comparison of placental PLGF **(A)** and VEGFR1 **(B)** concentration in NPD, PD and PE animals at the end of the experimental period. Values are presented as means and vertical bars indicate SD (*n* = 6 in each group). ^β^*p* < 0.05 vs. NPD and ^μ^*p* < 0.05 vs. PD.

Figure 3 shows the placental PLGF (Figure 3A) and VEGFR1 (Figure 3B) concentrations in NPD, PD, and PE animals at the end of the experimental period. A significant difference was observed between the experimental groups, as shown in Figure 3A [F_(2, 10)_ = 102.2, *p* = 0.0001] and Figure 3B [F_(2, 10)_ = 33.14, *p* = 0.0001]. The PD group had a significantly lower PLGF concentration in the placenta than the NPD group ^β^ (NPD vs. PD, *p* < 0.05, Figure 3A). Additionally, the PE group had a significantly lower PLGF concentration in the placenta than the NPD group ^β^ (NPD vs. PE, *p* < 0.05, Figure 3A). Interestingly, the PD group had a significantly higher PLGF concentration in the placenta than the PE group ^μ^ (PD vs. PE, *p* < 0.05, Figure 3A). Furthermore, the PD group had a significantly lower VEGFR1 concentration in the placenta than the NPD group ^β^ (NPD vs. PD, *p* < 0.05, Figure 3B). Additionally, the PE group had a significantly lower VEGFR1 concentration in the placenta than the NPD group ^β^ (NPD vs. PE, *p* < 0.05, Figure 3B). Interestingly, the PD group had a significantly higher VEGFR1 concentration in the placenta than the PE group ^μ^ (NPD vs. PE, *p* < 0.05, Figure 3B).

### 3.5. Placental TNFα and IL-6 gene expression and placental IFN- γ concentration

**Figure 4:**
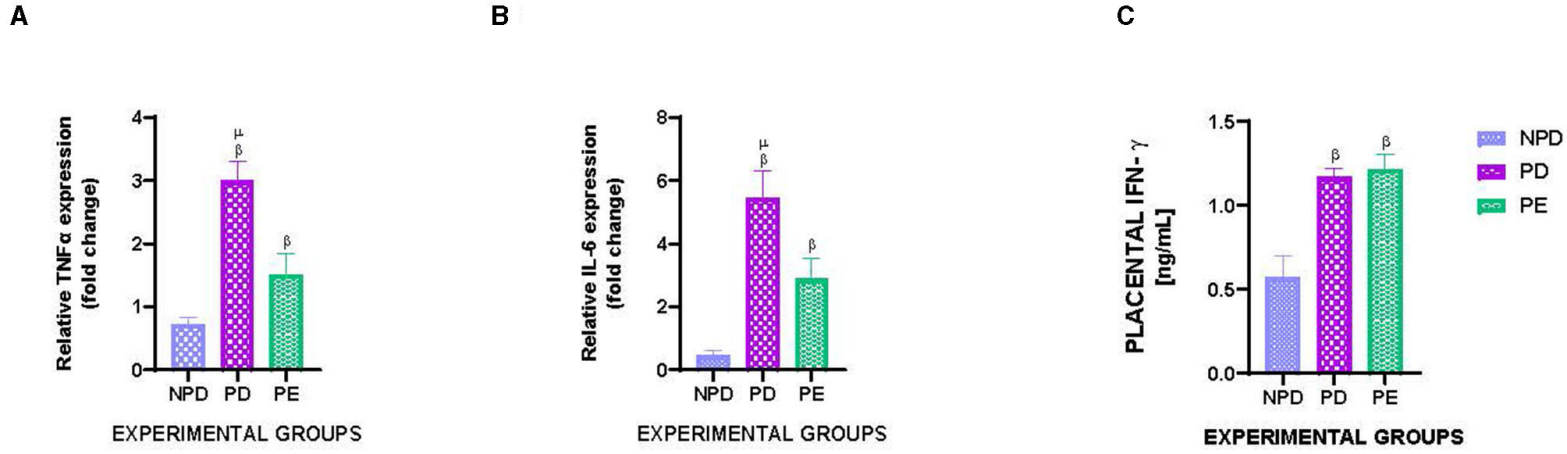
Placental TNFα and IL-6 gene expression and placental IFN- γ concentration. Comparison of mRNA expression of placental TNFα **(A)** and IL-6 **(B)** and placental IFN- γ concentration **(C)** in NPD, PD and PE animals at the end of the experimental period. Values are presented as means and vertical bars indicate SD (*n* = 6 in each group). ^β^*p* < 0.05 vs. NPD and ^μ^*p* < 0.05 vs. PD.

Figure 4 shows the mRNA expression of placental TNFα (Figure 4A) and IL-6 (Figure 4B) and placental IFN- γ concentration in NPD, PD, and PE animals at the end of the experimental period. A significant difference was observed between the experimental groups, as shown in Figure 4A [F_(2, 10)_ = 86.22, *p* = 0.0001], Figure 4B [F_(2, 10)_ = 124.6, *p* = 0.0001], and Figure 4C [F_(2, 10)_ = 104.4, *p* = 0.0001]. The PD group had a significantly higher TNFα expression than the NPD group ^β^ (NPD vs. PD, *p* < 0.05, Figure 4A). Additionally, the PE group had a significantly higher TNFα expression than the NPD group ^β^ (NPD vs. PE, *p* < 0.05, Figure 4A). Interestingly, the PD group had a significantly higher TNFα expression than the PE group ^μ^ (PD vs. PE, *p* < 0.05, Figure 4A). Furthermore, the PD group had a significantly higher IL-6 expression than the NPD group ^β^ (ND vs. PD, *p* < 0.05, Figure 4B). The PE group had a significantly higher IL-6 expression than the NPD group ^β^ (NPD vs. PE, *p* < 0.05, Figure 4B). Interestingly, the PD group had a significantly higher IL-6 expression than the PE group ^μ^ (PD vs. PE, *p* < 0.05, Figure 4B). In addition, the PD group had a significantly higher IFN- γ concentration in the placenta than the NPD group ^β^ (NPD vs. PD, *p* < 0.05, Figure 4C). Furthermore, the PE group had a significantly higher IFN- γ concentration in the placenta than the NPD group ^β^ (NPD vs. PD, *p* < 0.05, Figure 4C).

### 3.6. Plasma nitric oxide concentration

**Figure 5:**
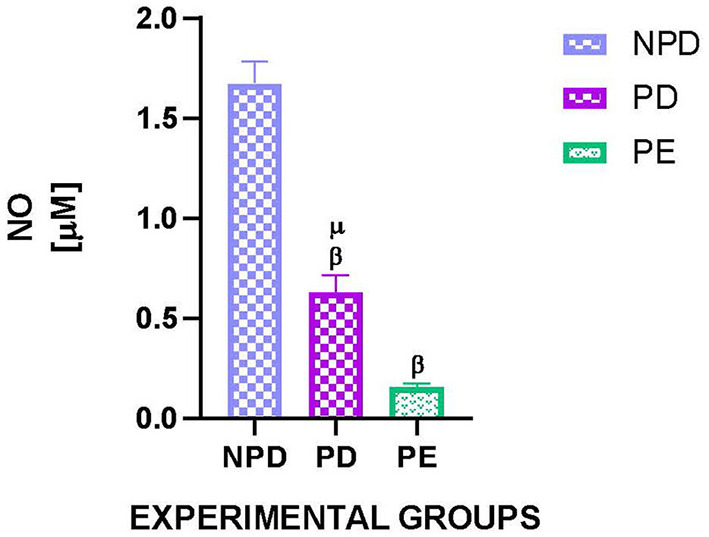
Plasma nitric oxide concentration. Comparison of plasma NO concentration in NPD, PD and PE animals at the end of the experimental period. Values are presented as means and vertical bars indicate SD (*n* = 6 in each group). ^β^*p* > 0.05 vs. NPD and ^μ^*p* < 0.05 vs. PD.

Figure 5 shows the plasma nitric oxide (NO) concentration of NPD, PD, and PE animals at the end of the experimental period. A significant difference was observed between the experimental groups [F_(2, 10)_ = 628.8, *p* = 0.0001]. The PD group had a significantly lower NO concentration than the NPD group ^β^ (NPD vs. PD, *p* < 0.05, Figure 5). Additionally, the PE group had a significantly lower NO concentration than the NPD group ^β^ (NPD vs. PE, *p* < 0.05, Figure 5). Furthermore, the PD group had a significantly higher plasma NO concentration than the PE group ^μ^ (PD vs. PE, *p* < 0.05, Figure 5).

## 4. Discussion

DM has been shown to be associated with a malfunctioning placenta, thus increasing the risk of the development of preeclampsia; therefore, the current study investigated the risk of the development of preeclampsia in animals with a history of prediabetes preconception. In our laboratory, we have designed an HFHC diet-induced prediabetic animal model (23, 30).

Hyperglycemia in DM increases the risk of developing PE; however, prediabetes is characterized by intermediate hyperglycemia which may also increase the risk of developing PE (31). PD animals maintained a high blood glucose concentration throughout the gestational period, which suggests that the PD animals had impaired glucose tolerance. Previous studies have also shown that impaired glucose tolerance is associated with insulin resistance and endothelial dysfunction (32, 33).

Hyperglycemia increases the production of a myriad of metabolic insults resulting in insulin resistance in the placenta which is accompanied by the stimulation of the transcription and expression of proinflammatory cytokines, thus promoting apoptosis and malfunction in the placenta (34). There is a lack of studies assessing placental inflammation in prediabetic conditions; however, Picchi et al. have shown the overexpression of TNFα and an impaired vascular bed, thus inducing endothelial dysfunction in prediabetic mice which is in line with our observations (35). At the end of the experimental period, PD animals showed an increase in the expression of placental IFN- γ, TNFα, and IL-6 in comparison to the NPD animals. Furthermore, IFN- γ, TNFα, and IL-6 have been shown to induce hypoxia which promotes endothelial dysfunction; however, the mechanisms are not fully established. Meanwhile, it is speculated that IFN- γ is associated with the suppression of anti-inflammatory cytokines such as IL-10 and further promotes inflammation (36). In addition, hypoxia has been shown to promote the production of sFLT in the placenta (37, 38). Furthermore, sFLT binds to the proangiogenic factor, PLGF, thus reducing its bioavailability to bind to VEGFR1 in the placenta (39, 40). In addition, sustained uncontrolled hyperglycemia has been shown to reduce PLGF production and VEGFR1 expression in the placenta, thus hindering angiogenesis and the establishment of a functional placental vascular network; however, the mechanism is not fully established (41). Interestingly, at the end of the experimental period, PD animals showed a decline in the proangiogenic factor PLGF in the placenta, which was associated with a decline in its receptor, VEGFR1, in comparison to the NPD animals. These observations are consistent with previous findings which suggest that prediabetes may induce hypoxia and promote placental malformation and malfunction. However, the sFLT concentration was not investigated in the current study, and further investigations are needed to outline this mechanism. In addition, Rätsep et al. showed that mice that are deficient in PLGF have abnormal placental vascularization and lack uniformity of the vessels formed (42). Furthermore, the decline in placental PLGF and VEGFR1 was worse in PE animals than in PD animals, suggesting that the establishment of the functional placental vasculature may be worse under preeclamptic conditions. L-NAME has been shown to dysregulate trophoblastic cell function by reducing the ability of the cells to migrate, which may contribute to the malformation of the placenta accompanied by the reduced PLGF and VEGFR1; however, the full mechanism is not well established (43).

Circulating proinflammatory cytokines have been shown to enter the maternal circulation; specifically, TNFα has been shown to reduce NO which is a vasodilator that maintains vascular homeostasis (44). TNFα has also been shown to downregulate the expression of endothelial NO synthase (eNOS) while increasing NF-κB at the mRNA level (45, 46). This results in the increased expression of adhesion molecules and chemokines which promote inflammation (47). Furthermore, the insulin signaling pathway has been shown to upregulate the expression of eNOS and NO; however, TNFα has been implicated in the downregulation of autophosphorylation and expression of some of the proteins in the insulin signaling pathway, such as the insulin receptor, thus inducing insulin resistance and chronic vasoconstriction (48). However, in the current study, the proteins in the insulin pathway were not investigated. Interestingly, our results are in agreement with the literature as the NO concentration in PD animals was lower compared with the NPD animals at the end of the experimental period. As expected, at the end of the experimental period, NO concentration in PE animals was lower than in the NPD animals. This is due to the reduction of eNOS activity by L-NAME, thus reducing NO production and bioavailability and promoting and maintaining vasoconstriction (49). At the end of the experimental period, NO concentration in PE animals was lower than in the PD animals, suggesting that the intermediate hyperglycemia may have not exhausted the eNOS enzymes, thus stimulating the production of NO. NO reduction observed in our PD animals was also associated with high MAP compared with the NPD animals, as vascular homeostasis was disturbed and vasoconstriction may have been maintained. Thus, PD is associated with endothelial dysfunction which is also observed in PE. As predicted, PE animals also showed a high MAP as the L-NAME induced vasoconstriction and endothelial dysfunction. Current observations are in line with previous studies that confirm the increase in blood pressure observed in L-NAME-induced hypertensive rats as a preeclamptic animal model (50).

Increased MAP is associated with elevated peripheral resistance and endothelial dysfunction that may be associated with hypoxia in the placenta which compromises the nutrient supply to the developing fetus (51). In the current study, PD animals had slightly heavier placentas and increased fetoplacental ratios, which was inversed in PE animals. Castillo-Castrejon et al. have demonstrated that hyperglycemia upregulates the expression and activity of nutrient transporters including the mechanistic target of rapamycin (mTOR) in trophoblasts, thus increasing placental and fetal weight (52). Intermediate hyperglycaemia therefore, may have increased nutrient transporters in the placenta resulting in fetal weight gain which may have been associated with macrosomia and IUGR which is commonly observed in preeclamptic patients. However, the mTOR pathway was not investigated in the current study. Furthermore, the endothelial dysfunction associated with LNAME may have also induced placental hypoxia, thus reducing the nutrient supply to the fetus. Hence, reduced placental weight and fetoplacental ratio were observed in preeclamptic animals. In agreement with current results, Rueda et al. also showed that LNAME is associated with poor embryo implantation and placentogenesis, consequently declining placental and fetal weight in rats (53).

## 5. Conclusion

There is limited literature exploring the risk of the development of PE in prediabetic patients. Hence, the current study explores PD as a risk factor for the development of PE. The current results show that PD induces impaired glucose tolerance and intermediate hyperglycemia associated with endothelial dysfunction and increased expression of proinflammatory cytokines. Increased proinflammatory cytokines may have reduced trophoblastic invasion and migration, consequently disrupting the establishment of a functional placental vasculature. Increased proinflammatory cytokines may have also reduced the expression of eNOS, reducing NO bioavailability and maintaining vasoconstriction; hence, a rise in MAP and endothelial dysfunction is observed in preeclamptic patients. The maintained increased MAP may have reduced sufficient supply to the placenta resulting in placental hypoxia, which further reduced fetal nourishment. Additionally, the intermediate hyperglycemia may have been shunted to lipid synthesis which may have resulted in an increased placental and fetal weight, which contributes to macrosomia and IUGR commonly observed in PE. However, further studies are required to explore other mechanisms that may increase the development of PE in prediabetic animals and patients. Current observations emphasize the need for prediabetes screening and monitoring during pregnancy, as prediabetes is a risk factor similar to DM, which predisposes the mother to preeclampsia.

## Data availability statement

The raw data supporting the conclusions of this article will be made available by the authors, without undue reservation.

## Ethics statement

The animal study was approved by Animal Research Ethics Committee, University of KwaZulu-Natal. The study was conducted in accordance with the local legislation and institutional requirements.

## Author contributions

AL, AS, NS, AK, and PN contributed to the conceptualization, design, and all formal analyses of the study. AL, AS, NX, AN, and PN contributed to the data acquisition of the study. AL, AK, NS, and PN contributed to the original writing and funding acquisition of the study. All authors contributed to the manuscript revision, read, and approved the submitted version.
